# Monoacylglycerol Lipase Inhibitor MJN110 Reduces Neuronal Hyperexcitability, Restores Dendritic Arborization Complexity, and Regulates Reward-Related Behavior in Presence of HIV-1 Tat

**DOI:** 10.3389/fneur.2021.651272

**Published:** 2021-08-16

**Authors:** Alexis F. League, Benjamin L. Gorman, Douglas J. Hermes, Clare T. Johnson, Ian R. Jacobs, Barkha J. Yadav-Samudrala, Justin L. Poklis, Micah J. Niphakis, Benjamin F. Cravatt, Aron H. Lichtman, Bogna M. Ignatowska-Jankowska, Sylvia Fitting

**Affiliations:** ^1^Department of Psychology and Neuroscience, University of North Carolina Chapel Hill, Chapel Hill, NC, United States; ^2^Department of Pharmacology and Toxicology, Virginia Commonwealth University, Richmond, VA, United States; ^3^Department of Chemistry, Scripps Research Institute, La Jolla, CA, United States; ^4^Okinawa Institute of Science and Technology, Neuronal Rhythms in Movement Unit, Okinawa, Japan

**Keywords:** endocannabinoids, excitotoxicity, HIV, Tat, monoacylglycerol lipase, MJN110, 2-arachidonoyl glycerol

## Abstract

While current therapeutic strategies for people living with human immunodeficiency virus type 1 (HIV-1) suppress virus replication peripherally, viral proteins such as transactivator of transcription (Tat) enter the central nervous system early upon infection and contribute to chronic inflammatory conditions even alongside antiretroviral treatment. As demand grows for supplemental strategies to combat virus-associated pathology presenting frequently as HIV-associated neurocognitive disorders (HAND), the present study aimed to characterize the potential utility of inhibiting monoacylglycerol lipase (MAGL) activity to increase inhibitory activity at cannabinoid receptor-type 1 receptors through upregulation of 2-arachidonoylglycerol (2-AG) and downregulation of its degradation into proinflammatory metabolite arachidonic acid (AA). The MAGL inhibitor MJN110 significantly reduced intracellular calcium and increased dendritic branching complexity in Tat-treated primary frontal cortex neuron cultures. Chronic MJN110 administration *in vivo* increased 2-AG levels in the prefrontal cortex (PFC) and striatum across Tat(+) and Tat(–) groups and restored PFC N-arachidonoylethanolamine (AEA) levels in Tat(+) subjects. While Tat expression significantly increased rate of reward-related behavioral task acquisition in a novel discriminative stimulus learning and cognitive flexibility assay, MJN110 altered reversal acquisition specifically in Tat(+) mice to rates indistinguishable from Tat(–) controls. Collectively, our results suggest a neuroprotective role of MAGL inhibition in reducing neuronal hyperexcitability, restoring dendritic arborization complexity, and mitigating neurocognitive alterations driven by viral proteins associated with latent HIV-1 infection.

## Introduction

With the advent of combination antiretroviral therapy (cART), mortality rates among human immunodeficiency virus type-1 (HIV-1)-infected individuals have decreased by more than 50% (1). The consequent growth in the population of people with latent HIV-1 (PWH) has introduced a new demand for supplemental treatments, as cART itself is neurotoxic with prolonged exposure (2, 3) and leads to greater susceptibility to issues driven by synaptic dysfunction including HIV-associated neurocognitive disorders [HAND, (4)], which occurs in up to 50% of infected individuals (5). Further, cART is largely unable to deplete expression of residual HIV-1 proteins in the tissues of the central nervous system [CNS; (6–9)]. One such viral protein, transactivator of transcription (Tat) enters the host genome early after infection (10), and has been shown to induce synaptodendritic injury and cognitive deficits in murine models of HIV-1 (11–14) by altering the cellular environment through proinflammatory processes which contribute significantly to the pathogenesis of HAND (7, 15, 16).

Previous work has demonstrated *in vitro* Tat excitotoxicity (17–19) which is downregulated in frontal cortex primary neuron cultures with direct application of endogenous ligands N-arachidonoylethanolamine (AEA) and 2-arachidonoylglycerol (2-AG) via cannabinoid receptors type-1 [CB_1_R; (20)]. Blocking enzymatic degradation of 2-AG and/or AEA likely has greater translational value, as activity of endogenous ligands and associated downstream products provides an extended therapeutic window due to longer half-life and greater conferred selectivity at target receptors relative to many currently available phytocannabinoid-based treatments (21–23). Additionally, therapeutic enhancement of cannabinoid signaling by enzyme inhibitors appears to be localized to sites of injury in contrast to direct agonists, which more widely affect cannabinoid signaling across the brain and are more likely to drive off-target effects (24–27).

The endocannabinoid system is a promising avenue for development of therapeutic strategies in disease, as existing literature shows anti-inflammatory and neuro-regulatory properties of agonists at CB_1_R (28–30) and cannabinoid receptors type-2 [CB_2_R; (31, 32)]. Potential neuroprotective effects of the endocannabinoid system in the context of neuroHIV have been reviewed previously (33, 34). Activation of CB_1_R and CB_2_R may downregulate the proinflammatory cytokine levels associated with synaptodendritic injury (35, 36), behavioral disturbances observed in PWH and HIV-1 transgenic rats (36, 37), and peripheral neuropathy (38–40). Nevertheless, therapeutic use of the CB_1_R agonists are limited due to associated pervasive psychoactive side effects including sensorimotor, affective, and cognitive disturbances (41). Thus, research efforts have focused on development of drugs targeting components of the endogenous cannabinoid system, including enzymes regulating the biosynthesis and degradation of the endogenous cannabinoids AEA and 2-AG to enhance tonic endocannabinoid activity (42–44).

Of particular interest is the effect of monoacylglycerol lipase (MAGL), which contributes to about 85% of total 2-AG hydrolysis in the CNS (45, 46). In addition to promoting activity at CB_1_R, inhibition of MAGL has recently been shown to downregulate inflammation in central (47) and peripheral (48) nervous system models by reducing breakdown of endogenous ligands into inflammatory metabolites such as arachidonic acid [AA; (49)] and downstream products like prostaglandins (47, 50). As increased prostaglandin activity drives inflammatory responses, reduction of AA production may reduce neuroinflammation caused by CNS insult. Indeed, MAGL inhibitor MJN110 has demonstrated neuroprotective effects in models of neuropsychiatric and neurodegenerative diseases (51) and ischemic stroke (52).

The aims for this project were 4-fold: first, to characterize neuroprotective effects of MJN110 treatment against Tat-associated excitotoxicity in frontal cortex neuron cultures via live calcium imaging; second, to assess Tat- and MJN110-induced alterations to neuronal morphology via immunocytochemistry *in vitro*; third, to assess the effects of Tat and MJN110 treatment *in vivo* using a HIV-1 Tat transgenic mouse model (13, 53) of behavioral flexibility as an indicator of MJN110 efficacy in restoring prefrontal cortex function (54–56); and fourth, to characterize brain region-specific alterations to endocannabinoid-related protein expression as a function of Tat and MJN110 treatment via ultrahigh performance liquid chromatography tandem mass spectrometry.

## Materials and Methods

Experiments were conducted in accordance with the NIH *Guide for the Care and Use of Laboratory Animals*. All procedures were approved by the University of North Carolina at Chapel Hill Institutional Animal Care and Use Committee.

### Primary Neuron Cultures

Primary neuron cultures were derived from embryonic day 17 (E17) C57BL/6J mouse (Charles River, Raleigh, NC) frontal cortex and incubated as previously described (32). Briefly, brains were collected and frontal cortex tissue was dissected and minced. Neurons were isolated with 30-min incubation (37°C) in neurobasal medium (ThermoFisher Scientific, #21103049, USA) with 2.5 mg/mL trypsin, 0.015 mg/mL DNAse, 2% B27 (50X; ThermoFisher Scientific, #17504044, USA), 0.5 mM L-glutamine (ThermoFisher Scientific, #25030081, USA), 25 mM glutamate (Sigma-Aldrich, #604968, USA), and 1% penicillin-streptomycin (ThermoFisher Scientific, #15140122, USA). Tissue was triturated and filtered twice through 70 μm pore nylon mesh before dissociated cells were plated on poly-L-lysine-coated (Sigma-Aldrich, #P2636) 35 mm glass-bottom dishes (MatTek, #P35G-0-10-C, USA; 1 ^*^ 10^5^ cells per dish) or cover slips (Fisherbrand 22 mm microscope cover slips, Cat No. 12-547, USA; 2 ^*^ 10^5^ cells per slip) for calcium imaging or immunocytochemistry, respectively. Neurons were maintained in a humidified incubator with 5% CO_2_ at 37 °C (Eppendorf, Hauppauge, NY) in neurobasal medium supplemented with 25 μM glutamate, 2% B27, 0.5 mM L-glutamine, and 1% penicillin-streptomycin. Supplemented medium was 50% exchanged every 48 h. On day *in vitro* 10, cells were prepared for imaging.

### Treatments *in vitro*

Primary frontal cortex neuron cultures were treated with HIV-1 Tat_1−86_ (50–100 nM; ImmunoDx, IIIB, #1002, USA), glutamate (0.1–10 μM; Sigma-Aldrich, #604968, USA), and/or MJN110 (0.5–1 μM; 50), which were diluted in Hanks Balanced Salt Solution (HBSS; ThermoFisher Scientific, #14025092, USA) supplemented with 10 mM HEPES (ThermoFisher Scientific, #15630080, USA). Tat_1−86_ concentrations in the 50–100 nM range were chosen for the present study as they recapitulate cellular deficits observed in PWH (57–60). For experiments using glutamate to induce excitation, a subthreshold concentration of Tat_1−86_ [50 nM; i.e., concentration insufficient to elicit excitatory response when bath-applied to neurons; established by (32) was used to drive neurons into a disease state prior application of glutamate during imaging. Concentrations of glutamate and MJN110 were chosen based on preliminary experiments (Supplementary Figure 1) and previous studies (61) which assessed activity elicited *in vitro* by this neurotransmitter and drug, respectively.

### Live-Cell Fluorescence Imaging

**Figure 1:**
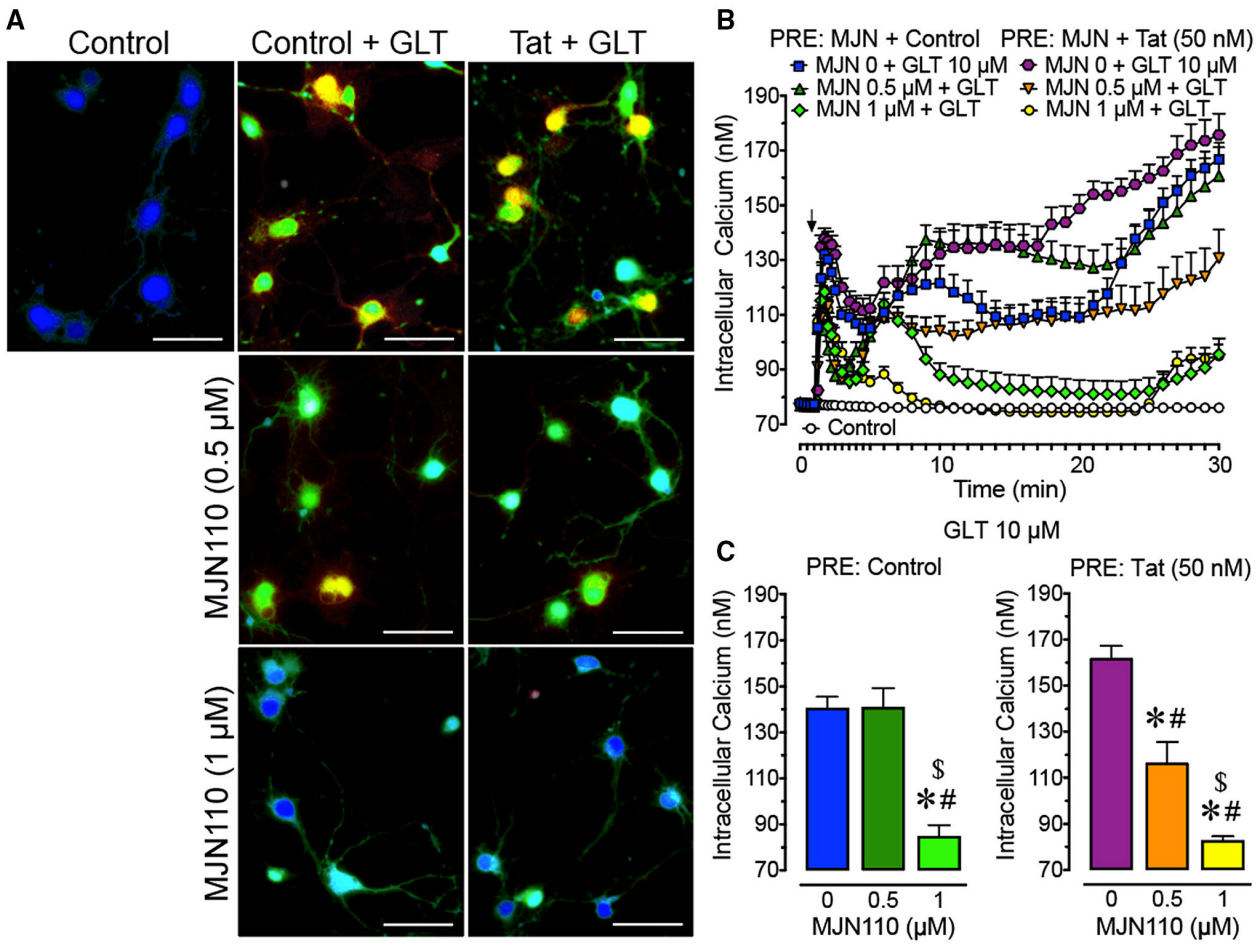
Primary frontal cortex neuron cultures (DIV 7-11) were untreated or pre-incubated with different concentrations of MJN110 (0–1 μM) and/or a subthreshold concentration of Tat 50 nM before Ca^2+^ imaging began (1 h and 30 min prior, respectively). **(A)** Pseudocolor images of neuronal ratiometric calcium imaging taken 30 min after a glutamate (GLT) 10 μM challenge (except for the control condition) with comparing frontal cortex neurons pre-incubated with vehicle solution or different concentrations of MJN110 (0.5–1 μM) and/or a subthreshold concentration of Tat 50 nM. **(B)** [Ca^2+^]_i_ levels were plotted over a 30-min time period with GLT 10 μM being applied at the 1-min mark (arrow). Application of GLT 10 μM onto neurons caused significant increases in [Ca^2+^]_i_ levels in the presence and absence of Tat and this effect was inhibited with MJN110 pretreatment in a concentration dependent manner. **(C)** The [Ca^2+^]_i_ levels are summarized for the last 10 min of calcium assessment and indicate that the lower concentration of MJN110 (0.5 μM) is more inhibitory in the presence of Tat compared to the control condition. Data are mean ± SEM. Statistical significance was determined using ANOVA and Bonferroni correction where applicable. An alpha level of *p* < 0.05 was considered significant for all statistical tests. *p < 0.05 vs. GLT 10 μM (PRE: Control); ^#^*p* < 0.05 vs. GLT 10 μM (PRE: Tat 50 nM); ^$^*p* < 0.05 vs. MJN110 0.5 μM + GLT 10 μM (PRE: Control). GLT, glutamate; PRE, pretreatment.

**Figure 2:**
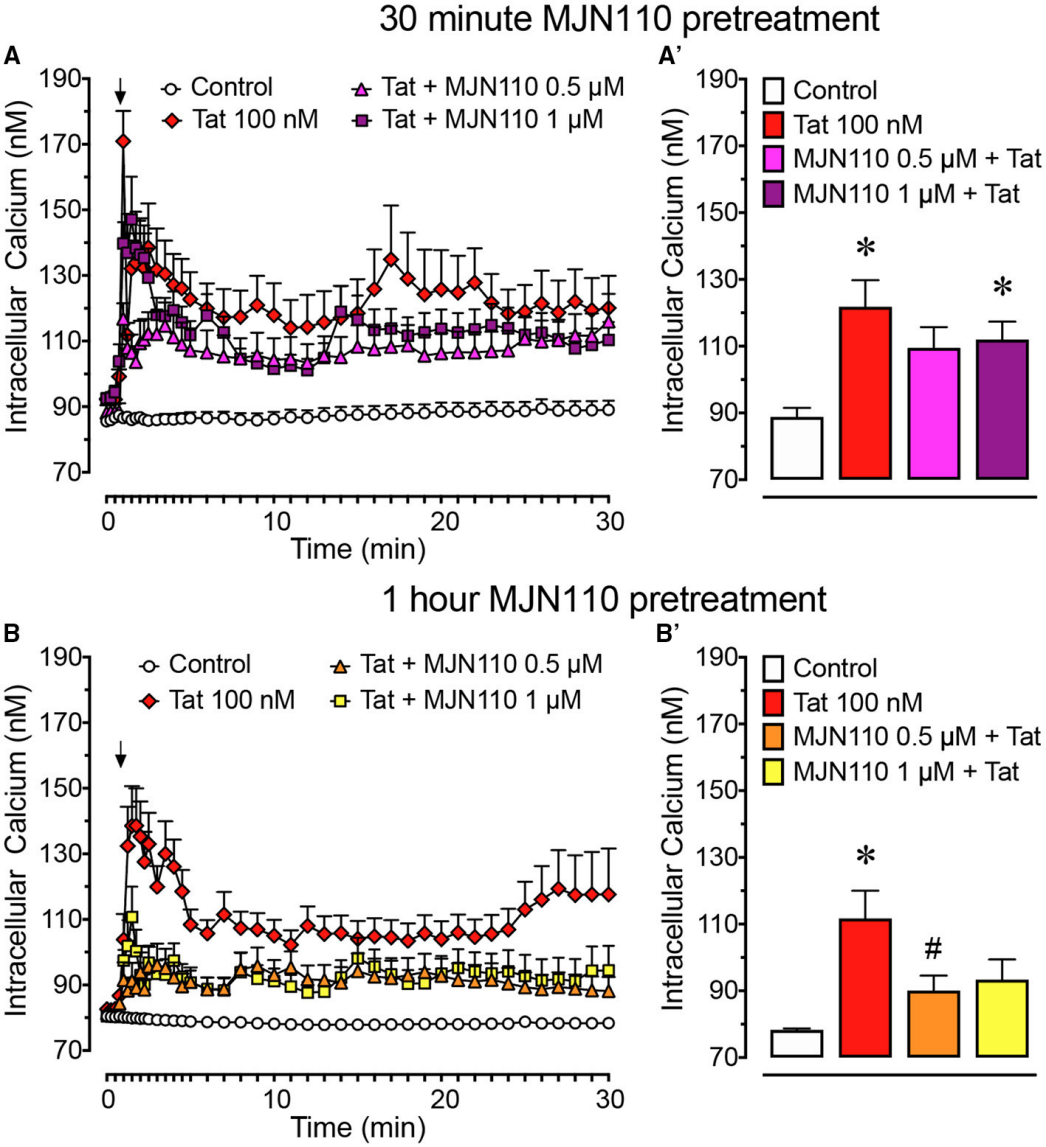
Primary frontal cortex neuron cultures (DIV 7-11) were untreated or pre-incubated for **(A)** 30 min or **(B)** 1 h with different concentrations of MJN110 before [Ca^2+^]_i_ imaging began. [Ca^2+^]_i_ levels were plotted over a 30-min time period with Tat 100 nM being applied at the 1-min mark (arrow). **(A)** Pre-incubation of MJN110 for 30 min prior to Tat application was not able to inhibit significant increases in [Ca^2+^]_i_ induced by Tat when observed across a 30-min time period. **(A')** The [Ca^2+^]_i_ levels are summarized for the last 10 min of calcium assessment and indicate that none of the MJN110 concentrations is able to inhibit Tat-induced increases in [Ca^2+^]_i_ levels. **(B)** Pre-incubation of MJN110 for 1 h prior to Tat application inhibited Tat-associated [Ca^2+^]_i_ upregulation over a 30-min time period. **(B')** The [Ca^2+^]_i_ levels are summarized for the last 10 min of calcium assessment and indicate that MJN110 0.5 μM was able to significantly inhibit Tat-induced [Ca^2+^]_i_ increases. Data are mean ± SEM. Statistical significance was determined using ANOVA and Bonferroni correction where applicable. An alpha level of *p* < 0.05 was considered significant for all statistical tests. *p < 0.05 vs. Control; ^#^*p* < 0.05 vs. Tat 100 nM.

Neurons were incubated for 30 min in fluorescent intracellular calcium indicator fura-2 AM (2 μL/mL; ThermoFisher Scientific, #F1221, USA) diluted in HBSS (with Ca^2+^, ThermoFisher Scientific, #14025076, USA) supplemented with HEPES (10 mM; ThermoFisher Scientific, #15630080, USA) according to manufacturer instructions. Half of the neurons were then exposed to 50 nM Tat (Figure 1) and/or MJN110 (500 nM or 1 μM; Figure 2) for an additional 1 h or 30 min prior to imaging (Figures 1, 2, respectively). Relative fluorescence ratio images were recorded for 30 min with a computer-controlled stage encoder with environmental control (37 °C, 95% humidity, 5% CO_2_) using a Zeiss Axio Observer Z.1 inverted microscope (Zeiss, Thornwood, NY, USA) with a 20x objective at 340/380 nm and 510 nm excitation and emission wavelengths, respectively. Following 1 min baseline imaging, 10 μM glutamate (Figure 1) or 100 nM Tat (Figure 2) was bath-applied to cultures. Excitation patterns were assessed for the remaining 29 min. Fifteen neurons were randomly selected from each culture and somas from each were tagged as regions of interest. Relative fluorescence ratios were used to quantify fluctuations in intracellular calcium ion ([Ca^2+^]_i_) activity across the experimental timeframe (62). At least three independent experiments were run for each treatment group.

### Immunocytochemistry

Neurons were fixed for 10 min with 4% paraformaldehyde in phosphate-buffered saline (ThermoFisher Scientific, #J61899-AP, USA) and stained as previously described (32). In brief, neurons were immunolabeled using primary antibodies against MAP2ab (Millipore, MAB378, USA; 1:500) with secondary antibodies conjugated to goat-anti-mouse Alexa 488 (ThermoFisher Scientific, #O-6380, USA; 1:1,000) diluted in PBS (ThermoFisher Scientific, #20012043). Nuclei of cells were stained using Hoechst 33342 (3 min; ThermoFisher Scientific, #H3570, USA) and coverslips were mounted using Prolong Gold (ThermoFisher Scientific, #P36930, USA). Z-stack images were obtained using ZEN 2010 Blue Edition software (Zeiss, Thornwood, NY, USA) with a Zeiss LSM 700 laser scanning confocal microscope using a 63x immersion objective (Zeiss, Thornwood, NY, USA). Dendritic branching complexity (e.g., maximum process length and distance from soma with maximal branching) and soma area were assessed with orthogonal projections from Z-stack images using the Sholl analysis tool within ImageJ software [Version 2.1.0; (63)].

### Animals

Brain-restricted, doxycycline-inducible HIV-1 IIIB Tat_1−86_ transgenic mice were developed on a hybrid C57BL/6J background as previously described (53, 64) using a tetracycline “on” system. Mice expressing Tat under the tetracycline-responsive element were crossed with mice expressing glial fibrillary acidic protein (GFAP) promoter-driven reverse tetracycline transactivator. Expression was induced with 6 mg/g doxycycline (DOX) administration through chow diet (product TD.09282; Envigo, Indianapolis, IN, USA). Genotyping by PCR was performed at 4 weeks of age to determine which mice were Tat(+) (i.e., expressing both GFAP-rTA and TRE-tat genes) and which were Tat(–) (i.e., expressing only the GFAP-rtTA gene).

Twenty-four female transgenic mice [12 Tat(+)] 3–4 months of age were held on *ad libitum* DOX chow diet (6,000 ppm, TD.09282, Envigo, NJ, USA) for 3 months prior to and throughout behavioral testing to induce and maintain Tat expression. All tests took place in the colony room during the dark phase of the 12-h light cycle.

### Treatments *in vivo*

**Figure 3:**
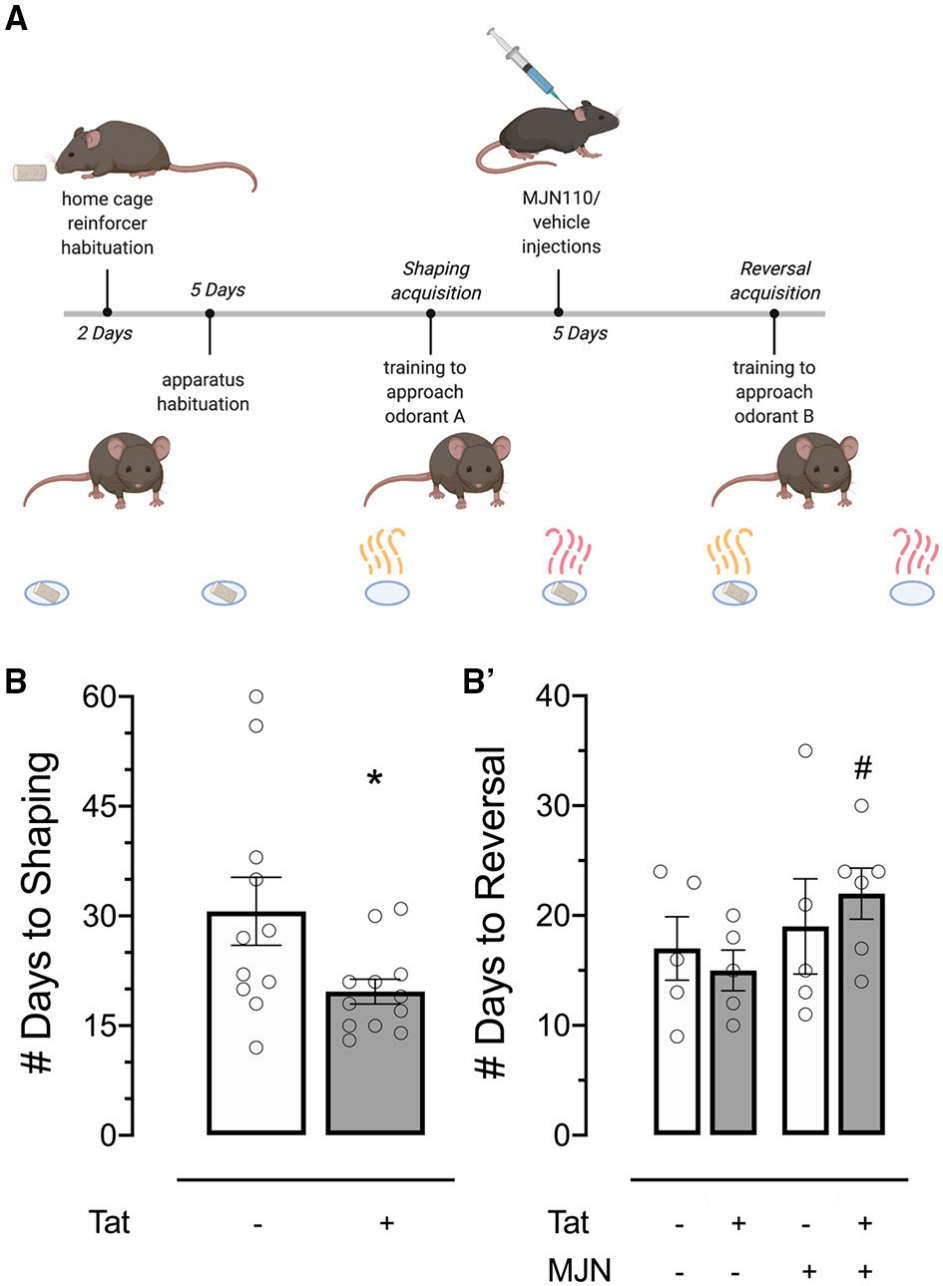
**(A)** Behavioral timeline schematic for the two-choice Odor Discrimination Flexibility (ODF) task. **(B)** Tat(+) subjects acquired the shaping task significantly faster than Tat(–) controls. **(B')** MJN110-treated Tat(+) subjects acquired the reversal task significantly slower than vehicle-treated Tat(+) subjects. Data are mean ± SEM. Statistical significance was determined using ANOVA and Bonferroni correction where applicable. An alpha level of *p* < 0.05 was considered significant for all statistical tests. *p < 0.05 vs. Tat(–); ^#^*p* < 0.05 vs. Tat(+)/vehicle.

For behavioral experiments, 1 mg/kg MJN110 (61) dissolved in saline-based vehicle [1:1:18; ethanol, Kolliphor (Sigma-Aldrich, #C5135, USA), and 0.9% NaCl saline, respectively; (25)] or vehicle alone was injected subcutaneously (10 μL/g body mass) for 5 days preceding, then throughout reversal trials (Figure 3). All injections were performed approximately 2 h before behavioral testing.

### Odor Discrimination Flexibility Task

#### Behavioral Assay

Mice were habituated to reinforcers (sweetened yogurt chips; Bio-Serv, Flemington, NJ, USA) and the test environment (3 min/day) for 7 and 5 days, respectively, preceding shaping trials (Figure 3A). Following habituation, two cups scented individually with 100 μL peanut oil (Amazon, #B00QGWM57M, USA) and 2-phenylethanol (2-PE; Sigma-Aldrich, #77861, USA) were placed at east and west ends of the test arena (Supplementary Figure 2, courtesy of G.F. League Co., Inc., Greenville, SC, USA), in recessed areas where reinforcers (quartered to reduce satiation) remained out of sight until a nose poke response was made. One reinforcer was available per trial.

Mice were trained 5 days per week in the two-choice operant paradigm wherein one olfactory stimulus was paired with the reinforcer (Figure 3A). Odorants were used at response sites to aid in stimulus discrimination (65) and mask any odor which may be present in reinforcers, which could otherwise bias response learning (66). Reward-paired scent was randomly assigned and counterbalanced across subjects, and target location was randomized between trials to preclude location-based learning. Experimenters were blind to subject genotype throughout behavioral testing and data analysis.

#### Shaping Trials

Subjects were placed into a holding chamber at the south end of the test arena. To signal a trial, the holding area was briefly (2 s) illuminated from above with a mildly aversive white LED light before the partition was lifted to cue access to the darker test arena, illuminated with red light. The white trial signal light remained on until subjects entered the test area or for 1 min of no entry, after which point subjects were manually directed to the arena from the holding chamber. Upon subject entry, the partition was closed and latency to interact with reward-paired odor location was recorded. Trials began when the subject body crossed into the testing area, and terminated upon reward consumption. All sessions were video-recorded (GoPro Hero6 Black; GoPro Smart Remote; Vanguard ESPOD CX1OS tripod) and analyzed by two experimenters to assess response latency and correctness (97.92% inter-rater agreement; Cohen's *k* = 0.79).

#### Drug Administration

After consistent discriminative choice for the cup paired with reward was established (i.e., a nose poke into the positive predictor cup and no interaction with the negative predictor cup across 8 out of 10 consecutive trials; 12–60 days), subjects were injected subcutaneously as described above. Shaping trials were continued during drug habituation to maintain learned responses, and reversal training began on injection day 6 (Figure 3A).

#### Reversal Training

The reversal paradigm was identical to that of shaping, except the opposite scent predicted reward. Injections were administered daily throughout reversal training. After consistent discriminative choice for the opposite cup was established (using the same criteria for the acquisition phase; 9–35 days), the experiment was terminated and subjects advanced to protein quantification analysis with mass spectrometry. Within-trial response latency was recorded to assess potential locomotor deficits/cannabimimetic effects presenting as slower approach to a reward-predictive cue.

#### Reinforcer Consumption Test

Tat has previously been shown to induce reward deficits and increase sensitivity to reinforcer-induced reward enhancement, contributing to depressive and addictive phenotypes, respectively (67). To measure anhedonic response and assess whether genotype influences reward salience of the reinforcer used in the ODF task, a consumption test and olfactory sensitivity test was conducted with a separate cohort of mice in home cages. After 5 days habituation to reinforcers, subjects were given access to a large amount of reinforcer (1.35 g) for 5 min and total volume consumed was quantified by measuring change in reinforcer weight.

#### Olfactory Sensitivity Test

Olfaction abilities were probed to ensure genotype-dependent differences in acquisition latency were not due to greater sensitivity of one group in detecting reinforcer odor in the ODF task. In this task, a reinforcer was buried in the center of home cages 0.5 cm beneath the bedding surface (68). Subjects were placed inside the south end of the cage, and latency to locate and consume the reinforcer was recorded. Trials terminated upon reinforcer consumption or after 5 min, whichever occurred first.

### Ultraperformance Liquid Chromatography/Tandem Mass Spectrometry (UPLC-MS/MS)

Subjects were sacrificed by rapid decapitation following isoflurane-induced anesthesia and brains were collected, dissected, and snap-frozen in liquid nitrogen. Calibration curves were prepared at the following concentrations: 0.028 pmol to 2.8 pmol for N-arachidonoylethanolamine (anandamide; AEA), 2.6 pmol to 260 pmol for 2-arachidonoylglycerol (2-AG), 0 and 0.033 nmol to 3.3 nmol for AA along with negative and blank controls. Samples were stored at −80 °C until the day of analysis. The internal standard (ISTD) was added to each calibrator, control, and sample except the blank control at concentrations of 0.28 pmol AEA-d8, 26 pmol 2-AG-d8, 0 and 0.33 nmol AA-d8. The calibrator, control and samples were analyzed as previously described (69). In brief, samples were homogenized in 100 μL ethanol and then 900 μL water was added. Sample cleanup was performed using UCT Clean Up® C18 solid phase extraction column (United Chemical Technologies, Inc., Bristol, PA, USA) conditioned with methanol followed by water. Samples were added and the columns were then washed with deionized water. Lipids were eluted with methanol, evaporated under nitrogen, and reconstituted in mobile phase. A Shimadzu UPLC system (Kyoto, Japan) attached to a Sciex 6500 QTRAP system with an IonDrive Turbo V source for TurbolonSpray® (Sciex, Ontario, Canada) controlled by Analyst software (Sciex, Ontario, Canada) was used for the analysis of AEA, 2-AG, and AA.

Chromatographic separation of AEA, 2-AG, and AA was performed on a Discovery® HS C18 Column 15 cm × 2.1 mm, 3 μm (Supelco: Bellefonte, PA, USA) kept at 25°C. The mobile phase consisted of A: acetonitrile and B: water with 1 g/L ammonium acetate and 0.1% formic acid. The following gradient was used: 0.0–2.4 min at 40% A, 2.5–6.0 min at 40% A, hold for 2.1 min at 40% A, then 8.1–9 min 100% A, hold at 100% A for 3.1 min and return to 40% A at 12.1 min with a flow rate of 1.0 mL/min. The source temperature was 600°C with ionspray voltage of 5,000 V. The curtain gas and source gases 1 and 2 had flow rates of 30, 60, and 50 mL/min, respectively. The mass spectrometer was operated in multiple reaction monitoring (MRM) positive ionization mode for AEA, 2-AG, and negative ionization mode for AA. The following transition ions (m/z) with their corresponding collection energies (eV) in parentheses were measured as follows: AEA: 348>62 (13) and 348>91 (60); AEA-d8: 356>63 (13); 2-AG: 379>287 (26) and 379>296 (28); 2-AG-d8: 384>287 (26); AA: 303>259 (-25) and 303>59 (-60); AA-d8: 311>267 (-25). The total run time for the analytical method was 14 min. Calibration curves were analyzed with each analytical batch for each analyte. A linear regression of the ratio of the peak area counts of analyte and corresponding deuterated ISTD vs. concentration was used to construct calibration curves.

### Data Analysis

Mean [Ca^2+^]_i_ change time course data from *in vitro* experiments were analyzed using analysis of variance (ANOVA) when appropriate. Violations of compound symmetry in repeated-measures ANOVAs for the within-subjects factors (i.e., comparing time points) were addressed by using the Greenhouse-Geisser degrees (*p*_GG_) of freedom correction factor (70). Separate ANOVAs followed by Bonferroni *post-hoc* analysis were conducted for the final 10 min of the experimental time course to assess differences in sustained excitation between treatment groups.

Behavioral data for the shaping phase are plotted as latency (days) required to meet advancement criteria, and were analyzed as survival curves using the logrank test. Behavioral data for the reversal phase are plotted as latency (days) to meet completion criteria and latency (seconds) to meet criteria within trials, and were analyzed using Cox regression and two-way ANOVAs with genotype [2 levels: Tat(–) mice, Tat(+) mice] and MJN110 treatment (2 levels: vehicle, MJN110 1 mg/kg) as between-subjects factors where appropriate followed by Bonferroni *post-hoc* tests.

Brain region-specific endocannabinoid levels were analyzed by two-way ANOVAs with genotype [2 levels: Tat(–) mice, Tat(+) mice] and MJN110 treatment (2 levels: vehicle, MJN110 1 mg/kg) as between-subjects factors followed by Bonferroni *post-hoc* tests and correlated with mean within-trial response latency for the reversal phase.

All data are presented as mean ± SEM. Alpha values of < 0.05 were considered significant for all statistical tests. All experiments and data analyses were carried out by experimenters blind to treatment conditions.

## Results

### Live-Cell Fluorescence Imaging

#### Glutamate-Induced Intracellular [Ca^2+^]_i_ Increase Was Dysregulated by Tat Pretreatment and Downregulated by MJN110 in a Concentration-Dependent Manner

To understand the role of MAGL inhibition and Tat in mediating neurotoxicity after a glutamate challenge, [Ca^2+^]_i_ responses of frontal cortex neuron cultures pretreated with Tat (50 nM) and MJN110 (0–1 μM) and then challenged with glutamate (10 μM) during imaging (Figure 1) were investigated. For excitation, various glutamate concentrations were tested (0.1–10 μM) to induce a sustained [Ca^2+^]_i_ response for 30 min in frontal cortex neurons (Supplementary Figure 1). A three-way mixed ANOVA was conducted with Tat application [2 levels: control, Tat 50 nM], MJN110 treatment (3 levels: vehicle, 0.5 μM, 1 μM) as between-subjects factors and time as a within-subjects factor. Results demonstrated a significant main effect for time [*F*_(40, 12, 400)_ = 61.6, *p*_GG_ < 0.001] and MJN110 [*F*_(2, 310)_ = 34.9, *p* < 0.001]. Further significant interactions were noted for time x MJN110 [*F*_(80, 12, 400)_ = 16.6, *p*_GG_ < 0.001], time x Tat x drug [*F*_(80, 12, 400)_ = 4.7, *p*_GG_ < 0.001], and Tat x MJN110 [*F*_(2, 310)_ = 7.2, *p* = 0.001] (Figure 1B). A two-way ANOVA with Tat and MJN110 treatments as between-subjects factors was conducted on the last 10 min of the experimental time course and revealed a significant MJN110 effect [*F*_(2, 310)_ = 29.0, *p* < 0.001] and Tat x MJN110 interaction [*F*_(2, 310)_ = 4.6, *p* = 0.010] with MJN110 significantly downregulating [Ca^2+^]_i_ levels in a concentration dependent manner. Only in the presence of Tat, MJN110 0.5 μM elicited a significant attenuation of the glutamate-induced [Ca^2+^]_i_ activity compared to MJN110-free vehicle application (*p* < 0.001). MJN110 0.5 μM did not significantly downregulate [Ca^2+^]_i_ levels compared to vehicle for control conditions when Tat was absent (Figure 1C).

#### Tat-Induced Dysregulation of [Ca^2+^]_i_ Increase Was Mitigated by Pretreatment With MJN110 in a Time- and Concentration-Dependent Manner

To understand the role of MAGL inhibition in Tat-mediated neurotoxicity, [Ca^2+^]_i_ responses of frontal cortex neuron cultures to Tat 100 nM when pretreated with vehicle or MJN110 (0.5–1 μM) 30 min or 1-h prior imaging (Figure 2) was investigated. Two-way mixed ANOVAs were conducted with treatment (4 levels: control, Tat 100 nM, MJN110 0.5 μM +Tat, MJN110 1 μM + Tat) as a between-subjects factor and time as a within-subjects factor. When MJN110 was applied to neuron cultures 30 min prior Tat 100 nM, results demonstrated a significant main effect for time [*F*_(40, 11, 760)_ = 9.3, *p*_GG_ < 0.001], a main effect of treatment [*F*_(3, 294)_ = 10.6, *p* < 0.001], and a time x treatment interaction [*F*_(120, 11, 760)_ = 2.4, *p* = 0.001] (Figure 2A). A one-way ANOVA conducted on the last 10 min revealed a significant treatment effect [*F*_(3, 294)_ = 5.5, *p* = 0.001], with Tat 100 nM treatment and pretreatment of MJN110 1 μM + Tat showing significantly higher [Ca^2+^]_i_ levels compared to the control condition (*p* = 0.001 and *p* = 0.036, respectively; Figure 2A'). The MJN110 0.5 μM + Tat condition was not significantly different from control, nor did it differ from the Tat and MJN110 1 μM + Tat groups; thus indicating 30-min pretreatment with MJN110 prior Tat 100 nM excitation is not sufficient to inhibit [Ca^2+^]_i_ levels in frontal cortex neuron cultures.

When MJN110 was pretreated 1 h prior Tat 100 nM exposure, a two-way mixed ANOVA demonstrated a significant main effect for time [*F*_(40, 11, 840)_ = 6.4, *p*_GG_ < 0.001], a main effect of treatment [*F*_(3, 296)_ = 17.0, *p* < 0.001], and a time x treatment interaction [*F*_(120, 11, 840)_ = 2.7, *p* < 0.001] (Figure 2B). A one-way ANOVA conducted on the last 10 min of the experimental time course revealed a significant treatment effect [*F*_(3, 296)_ = 6.0, *p* = 0.001], with only Tat 100 nM treatment displaying significantly higher [Ca^2+^]_i_ levels compared to the control condition (*p* < 0.001) and significantly differing from the MJN110 0.5 μM + Tat condition (*p* = 0.044; Figure 2B'). No other effect was noted to be significant. Thus, results suggest that MJN110 pretreatment for 1 h prior to Tat 100 nM excitation is able to inhibit [Ca^2+^]_i_ activity in frontal cortex neuron cultures.

### Immunocytochemistry

#### Dendritic Branching Complexity Was Increased in Tat-Exposed Frontal Cortex Neurons Treated With MJN110

Soma area (μm^2^), maximum process length (μm), and distance from soma with maximal branching (defined by radial distance from the center of the soma with maximum number of intersections) were analyzed to assess changes to neuronal morphology driven by Tat (100 nM) and/or MJN110 (1 μM, Table 1). A two-way ANOVA with Tat and MJN110 treatment as between-subjects factors for soma area displayed no significant effect and/or interaction for Tat or MJN110 treatment. Maximum process length was also not significantly altered by MJN110, but trended toward decreased length in neurons treated with Tat (*p* = 0.070, Table 1). Distance from soma with maximal branching was significantly increased with MJN110 treatment and displayed a significant Tat x MJN110 treatment interaction such that Tat-untreated neurons showed no significant branch pattern differences with MJN110 treatment (*p* = 0.810), but Tat-treated neurons displayed significant increases in branching complexity with MJN110 treatment (*p* = 0.002, Table 1).

**Table 1:** Effects of Tat (100 nM) and MJN110 (1 μM) on neuronal morphology from frontal cortex neuron cultures ^a^.

**Measure**	**Tat**	**Vehicle**	**MJN110 (1 μM)**	**Tat effect**		**MJN110 effect**		**Tat x MJN110**	
		**Mean ± SEM**	**Mean ± SEM**	***F_**1, 32**_***	***p***	***F_**1, 32**_***	***p***	***F_**1, 32**_***	***p***
Soma area (μm^2^)	Control	174.6 ± 20.37	174.5 ± 13.86	<1.0	0.77	<1.0	0.93	<1.0	0.93
	Tat	179.8 ± 41.10	184.7 ± 21.00						
Maximum process length (μm)	Control	67.8 ± 2.55	72.3 ± 5.01	3.5	0.07	1.8	0.19	<1.0	0.81
	Tat	59.0 ± 4.72	65.6 ± 3.74						
Distance from soma with maximal branching	Control	26.1 ± 2.32	28.9 ± 2.32	<1.0	0.38	**11.6**	** <0.01**	**4.6**	**0.04**
	Tat	19.4 ± 1.94	31.7 ± 2.21						

a *Sholl analysis of neuronal morphology in frontal cortex neuron cultures in vehicle- or MJN110-treated control or Tat-treated neurons expressed as the mean ± SEM. The parameters measured by Sholl analysis are indicated in the first column. One-way ANOVAs for each dependent measure were conducted with Tat and MJN110 treatment as between-subjects factors. F-values and p-values are presented from ANOVA results. Bolded values denote significant differences at α = 0.05; mean ± SEM, n = 9 cells per group*.

### Odor Discrimination Flexibility Task

#### Rate of Shaping Acquisition Was Faster in Tat(+) Subjects

Behavioral acquisition in the shaping phase of the ODF task was analyzed to assess whether genotype affected the rate of task learning. Shaping acquisition was significantly faster in Tat(+) relative to Tat(–) subjects [Figure 3B; *X*${}_{\left(1,\text{N}=23\right)}^{2}$ = 6.422, *p* = 0.011].

#### Faster Reversal Acquisition in Tat(+) Subjects Was Slowed to Rates Comparable to Tat(–) Controls With MJN110 Treatment

Reversal acquisition latency was separately assessed to determine the effects of genotype and MJN110 treatment specifically on cognitive flexibility. While the effect of genotype demonstrated significance in omnibus tests of behavioral acquisition in the reversal phase [*X*${}_{\left(3,\text{N}=21\right)}^{2}$ = 7.983, *p* = 0.046], it was found to be statistically insignificant when treatment and its interaction with genotype were taken into account (Table 2). Specifically, within Tat(+) subjects, MJN110 treatment significantly increased the number of trials required to acquire the reversal learning task (Figure 3B'; 22.00 ± 2.32 vs. 15.00 ± 1.84 for MJN110- and saline-treated subjects, respectively; *p* = 0.048) presenting as latencies more similar to Tat(–) subjects.

**Table 2:** Effects of genotype and MJN110 treatment on latency (days) to acquire the reversal phase of the ODF task ^b^.

**Variables in the equation**	**B**	**SE**	**Wald**	**df**	**Sig**.	**Exp(B)**	**95% CI for Exp(B)**	
							**Lower**	**Upper**
Genotype	−0.624	0.598	1.088	1	0.297	0.536	0.166	1.731
Treatment	0.428	0.612	0.489	1	0.485	1.534	0.462	5.092
Genotype*Treatment	−1.328	0.949	1.959	1	0.162	0.265	0.041	1.702

b *Cox regression with genotype and treatment as factors. While omnibus tests found a significant effect of genotype in reversal learning, this effect loses significance when treatment and its interaction with Tat are factored into the model*.

#### Neither Genotype Nor MJN110 Treatment Significantly Affected Within-Trial Response Latency

**Figure 4:**
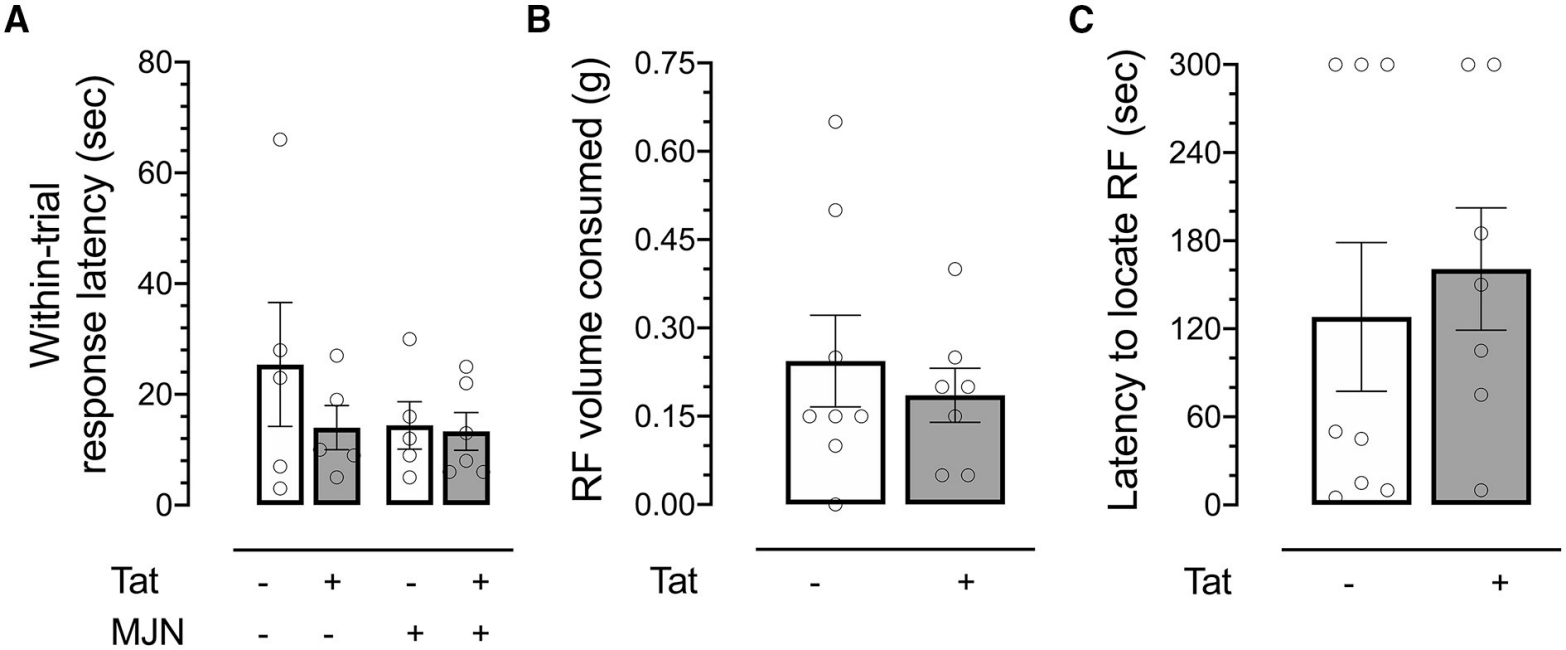
**(A)** No significant differences were observed between groups in response latency within reversal trials. **(B)** No significant differences in volume of reinforcer consumed were observed between genotypes. **(C)** No significant differences were observed between Tat(+) and Tat(–) subjects in latency to locate a hidden reinforcer. Data are mean ± SEM. Statistical significance was determined using ANOVA and Bonferroni correction where applicable. An alpha level of *p* < 0.05 was considered significant for all statistical tests. RF, reinforcer.

Response latency within trials was also assessed to determine whether genotype or drug treatment affected the speed with which subjects approached the reward-predictive cue. No significant effects of Tat or MJN110 on within-trial response latency were observed (Figure 4A; *p* = 0.337 and 0.368, respectively), indicating locomotor deficits/cannabimimetic effects were not likely factors driving observed differences.

#### Tat Expression Did Not Influence Reinforcer Consumption Volume

No significant differences were observed between Tat(–) and Tat(+) subjects in total volume consumed in the test session (Figure 4B), indicating the observed effect was not dependent upon appetite differences between groups.

#### Tat Expression Did Not Influence Olfactory Sensitivity

While previous work has demonstrated increased odor detection thresholds in HIV-positive relative to HIV-negative individuals (71), no differences in latency were observed between genotypes (Figure 4C), indicating the effect captured in the ODF task was not driven by genotype-associated differential sensitivity to odor.

### UPLC-MS/MS

#### 2-AG and AEA Were Differentially Expressed Across Examined Brain Regions Between Tat and MJN110 Conditions

**Figure 5:**
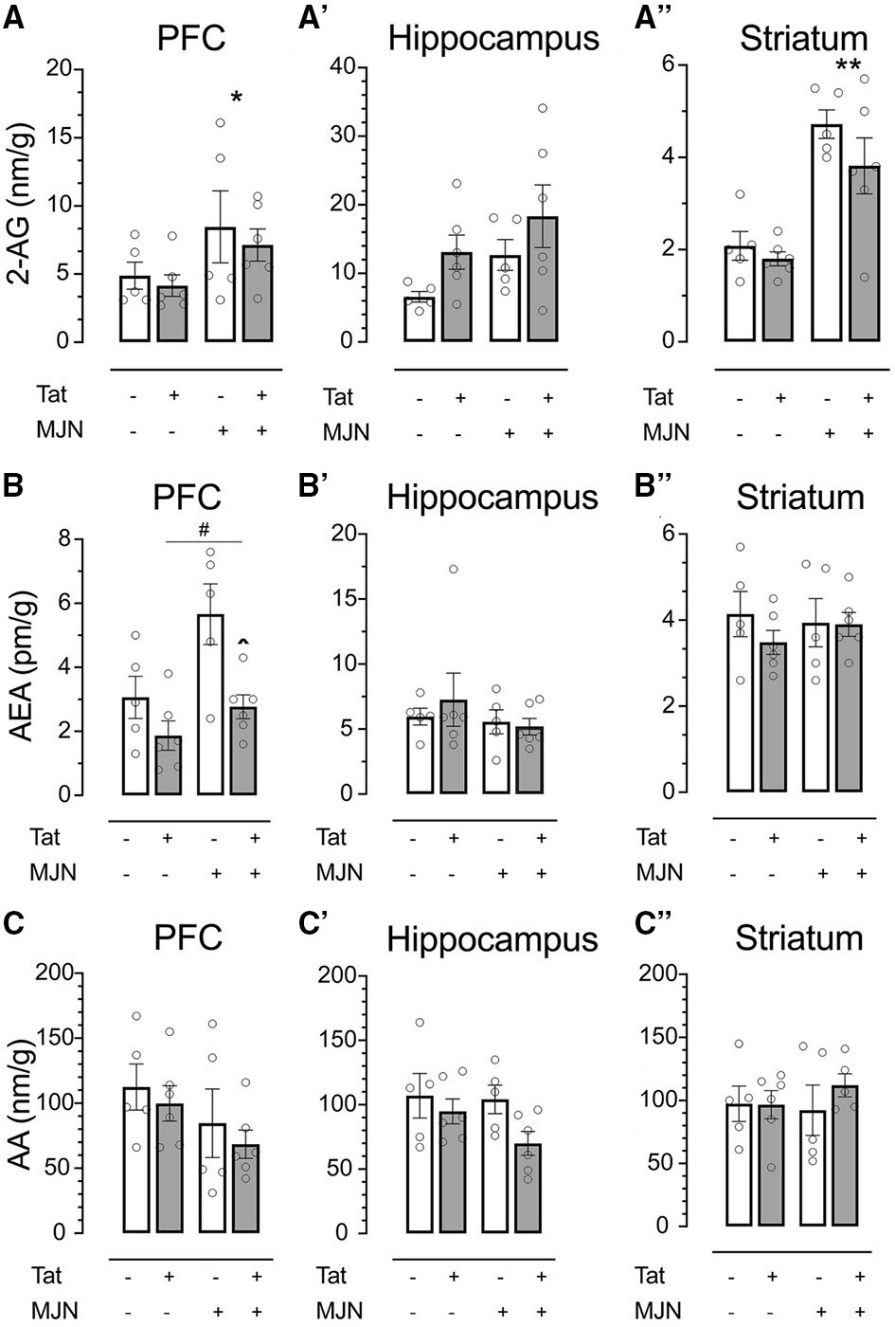
**(A)** MJN110 treatment significantly increased PFC 2-AG levels across genotypes. **(A')** Neither Tat nor MJN110 significantly affected hippocampal 2-AG levels. **(A”)** MJN110 significantly increased striatal 2-AG levels across genotypes. **(B)** Tat(+) subjects across treatment groups had significantly lower PFC AEA levels relative to Tat(–) controls. MJN110 significantly increased PFC AEA in Tat(+) subjects. No significant differences in hippocampal **(B')** or striatal AEA **(B”)** were observed between groups. No significant differences in PFC **(C)**, hippocampal **(C')**, or striatal AA **(C”)** were observed between groups. Data are mean ± SEM. Statistical significance was determined using ANOVA and Bonferroni correction where applicable. An alpha level of *p* < 0.05 was considered significant for all statistical tests. *p < 0.05 vs. vehicle treatment; **p < 0.0001 vs. vehicle treatment; ^∧^*p* < 0.05 vs. vehicle-treated Tat(+) subjects; ^#^*p* < 0.01 vs. Tat(−) subjects.

2-AG, AEA, and AA levels were quantified in the prefrontal cortex (PFC), hippocampus, and striatum to characterize the effects of genotype and MJN110 treatment on brain region-specific endocannabinoid levels. In vehicle-treated subjects, 2-AG levels across brain regions were not significantly affected by Tat (Figures 5A–A”; PFC *p* = 0.501, hippocampus *p* = 0.063, and striatum *p* = 0.155). However, MJN110 treatment significantly upregulated 2-AG in the PFC [Figure 5A; *F*_(1, 18)_ = 4.8, *p* = 0.042] and striatum [Figure 5A”; *F*_(1, 18)_ = 34.1, *p* < 0.0001]. While Tat(+) subjects had significantly lower PFC AEA levels relative to Tat(–) controls [*F*_(1, 18)_ = 11.0, *p* = 0.004], MJN110 significantly upregulated AEA in this region [Figure 5B; *F*_(1, 18)_ = 8.1, *p* = 0.011]. Neither hippocampal nor striatal AEA levels were significantly altered by MJN110 or Tat (Figures 5B',B”, respectively). No significant Tat- or MJN110-associated differences in AA levels were observed in any brain region assessed (Figures 5C–C”), though Tat(+) subjects trended toward lower hippocampal levels across treatments (Figure 5C'; *p* = 0.067).

#### PFC AA and Striatal AEA Levels Positively Correlated With Within-Trial Response Latency in Tat(+) and MJN110-Treated Subjects, Respectively

While neither genotype nor MJN110 treatment significantly affected PFC AA levels (*p* = 0.418 and *p* = 0.104, respectively), these measures were found to be positively correlated with response latency within behavior trials in Tat(+) subjects (*R*^2^ = 0.55, *p* = 0.014). A significant positive relationship was also found between striatal AEA levels and within-trial response latency across groups (*R*^2^ = 0.232, *p* = 0.031). This effect appears to be driven by MJN110, as drug-treated subjects displayed a stronger relationship than vehicle-treated controls (MJN110 *R*^2^ = 0.40, *p* = 0.050; vehicle *R*^2^ = 0.26, *p* = 0.136).

## Discussion

MJN110 treatment restored intracellular [Ca^2+^]_i_ response and dendritic branching complexity in Tat-treated neurons to that of vehicle-treated controls *in vitro*, and shifted reversal task acquisition latency among Tat(+) subjects to within the statistical range of Tat(-) controls *in vivo*. Given that the behavioral effect corresponded with significant Tat-induced and MJN110-induced increases in hippocampal, prefrontal cortex, and striatal 2-AG levels, the observed latency shift could be linked to treatment-dependent alteration of perceived reward salience.

Tat has been shown to induce neurotoxicity and synaptic damage across murine models of HIV, presenting as N-methyl-D-aspartate (NMDA) receptor phosphorylation, cytokine secretion, expression of apoptotic proteins, reduction of neurite length, and reduced appearance of puncta along neuronal processes (57, 72–75). Increasing 2-AG and AEA have previously been found to rescue effects of Tat in PFC neurons presenting as downregulation of high intracellular calcium levels and increased neuronal survival (20); further, 2-AG has a larger therapeutic window relative to AEA due to its higher physiological expression (76). Both 2-AG and MAGL are implicated in immune activation response control in macrophages and microglia, where 2-AG prevents proinflammatory cytokine production (77) and downregulates hippocampal inflammation-induced cyclooxegenase (COX)-2 expression in response to excitotoxic stimuli (78). MJN110-induced reduction of Tat-driven excitability is likely mediated by inhibitory effects of CB_1_R agonism *in vitro* as well as interactions with eicosanoid signaling pathways *in vivo* (79), though a CB_1_R knockout mouse model or co-administration of a CB_1_R antagonist such as rimonabant would be required to specifically delineate this potential mechanism.

While MJN110 and similar drugs have shown therapeutic potential in models of inflammation-associated neural dysfunction (32, 80), motivation regulation (81), stress (82), and neuropathic pain (83), beneficial aspects of these treatments may not generalize across test conditions. Elevation of 2-AG may regulate neural activity in subjects susceptible to excitotoxicity, but in a normal physiological context, the upregulation may result paradoxically in proinflammatory effects due to 2-AG hydrolysis into AA. AA metabolizes into other proinflammatory prostaglandins and eicosanoids (84) reported previously to be increased in women living with HIV (85). An interaction might exist between Tat(–) and Tat(+) subjects such that MJN110 treatment may shift activity of Tat(+) subjects closer to the level of Tat(–) untreated controls. MJN110 may have no additional beneficial effect in physiological systems wherein inhibitory correction is not needed. Alternative strategies may thus target upstream diacylglycerol lipase, responsible for biosynthesis of 2-AG (86). As MJN110 appears to drive different patterns of behavioral and neuronal activity and structure across physiological and pathological conditions, it could be explored whether depletion of 2-AG upstream might have greater neuroprotective potential as proinflammatory metabolites are further reduced and potential adverse effects of excessive 2-AG upregulation are functionally precluded (87, 88).

The finding that MJN110 (1 mg/kg) significantly upregulated AEA levels in the PFC of Tat(+) mice warrants further exploration. FAAH inhibition has shown to have brainregion-dependent effects on 2-AG levels (89), but the reverse effect for AEA with MAGL inhibition is less commonly found. While earlier-generation MAGL inhibitors such as JZL184 have some known cross-reactivity with FAAH, more selective MAGL inhibitors including MJN110 (25, 61) or KML29 (24, 90) show negligible cross-reactivity with FAAH and do not elevate AEA levels in the whole brain (24, 25, 61, 90). Similarly, we have not seen any AEA elevation in control, Tat(–) mice following repeated treatment with MJN110 at 1 mg/kg. It is likely Tat expression alone modifies endocannabinoid system function in such a way that MAGL blockade results in AEA elevation not observed in control animals, and the mechanism remains to be elucidated. It has been shown previously that Tat reduces the potency of 2-AG-induced inhibition on excitation (30).

HIV-1 has been shown to exert damage to dopaminergic cells and cause synaptic connectivity loss in dopaminergic projection pathways (91), presenting frequently in infected individuals as apathy and motivation dysregulation (92, 93). These behavioral sequelae of HAND are accompanied by increased markers of inflammation in the striatum (94). HIV-1 infection and substance abuse disorders are frequently comorbid (95, 96), and previous work has shown that neurons in regions implicated in reward seeking, such as the medial PFC, are hyperexcitable in the presence of HIV, particularly in models of salient reward self-administration (97, 98). As Tat binds and produces conformational changes to dopamine transporters (99, 100), rewarding effects of reinforcers are also influenced by direct Tat-induced inhibition of dopamine uptake in the striatum (101, 102). Specifically, Tat has been demonstrated to inhibit dopamine transporter (DAT) reuptake function by interacting (i.e., forming hydrogen bonds) with the DAT residues Tyr88 and His547 (103–106). Further, the resulting dopaminergic alterations can drive inflammation and immune dysfunction in PWH (107, 108) and increase susceptibility of these individuals to behavioral dysregulation presenting as greater addiction severity and HAND (109). Given the highly significant effect of MJN110 treatment on striatal 2-AG levels observed presently, subsequent investigations will shift focus to the effect of Tat and MJN110 on reward-seeking behavior as a proxy for motivation.

To better elucidate the effect of MJN110 on potential Tat-induced addiction-like behaviors (110), a progressive fixed ratio reinforcement schedule will be employed in an operant-conditioning task to assess reward-related motivation differences between genotypes and treatment groups. If data are consistent with Kesby et al. (67) and the effect of Tat is altered by MJN110 treatment, it is likely that the most influenced behavioral effect of the drug relies upon its action in cortico- and mesolimbic circuitry.

## Conclusion

As efforts continue to address shortcomings of currently available therapeutics in HIV-1 treatment, the present study aimed to characterize a potentially viable neuroprotective drug which has been shown to attenuate inflammatory responses across numerous models of CNS insult. Analyzing MAGL inhibition effects on Tat-induced behavioral, neuronal, and endocannabinoid level changes served as a proxy for understanding functional outcomes of chronic endocannabinoid signaling modulation, and whether targeting 2-AG at the stage of hydrolysis may be restorative in models of HAND. While the mechanistic actions and biological outcomes of novel cannabinoid drugs continue to be investigated, characterization of these compounds in disease states (particularly those which currently remain only partially suppressed) serves to broaden our understanding of their utility across models of inflammatory nervous system insult.

## Data Availability Statement

The raw data supporting the conclusions of this article will be made available by the authors, without undue reservation.

## Ethics Statement

The animal study was reviewed and approved by University of North Carolina at Chapel Hill Institutional Animal Care and Use Committee.

## Author Contributions

AFL, BLG, and SF wrote the manuscript. AFL, BLG, DJH, CTJ, BJY-S, JLP, and SF contributed to data collection and analysis. AFL, DJH, IRJ, BLG, JLP, MJN, BFC, AHL, BMI-J, and SF provided contributions to study design. All authors contributed to the article and approved the submitted version.

## Conflict of Interest

The authors declare that the research was conducted in the absence of any commercial or financial relationships that could be construed as a potential conflict of interest.

## Publisher's Note

All claims expressed in this article are solely those of the authors and do not necessarily represent those of their affiliated organizations, or those of the publisher, the editors and the reviewers. Any product that may be evaluated in this article, or claim that may be made by its manufacturer, is not guaranteed or endorsed by the publisher.
