# Supplementary material for: Monoacylglycerol Lipase Inhibitor MJN110 Reduces Neuronal Hyperexcitability, Restores Dendritic Arborization Complexity, and Regulates Reward-Related Behavior in Presence of HIV-1 Tat
Source: Front Neurol. 2021 Aug 16;12:651272. doi: 10.3389/fneur.2021.651272 (PMC8415271; doi:10.3389/fneur.2021.651272)

Supplemental Material

Supplemental Results

Calcium imaging

Tat pretreatment increases glutamate-induced $[Ca^{2+}]_i$ production in frontal cortex neuron cultures

To assess Tat's effects on glutamate-induced $[Ca^{2+}]_i$ production, we investigated $[Ca^{2+}]_i$ responses of frontal cortex neuron cultures pretreated with Tat (50 nM) and then challenged with glutamate (0.1 – 10 µM) during imaging (**Supplemental Figure 1**). A three-way mixed ANOVA demonstrated a significant main effect for time [$F(40, 10560) = 69.7$, $p_{GG} < 0.001$, Tat [$F(2, 264) = 12.0$, $p < 0.001$], and GLT [$F(2, 264) = 240.1$, $p < 0.001$]. Further significant interactions were noted for time x Tat [$F(80, 10560) = 6.3$, $p_{GG} < 0.001$], and time x GLT [$F(80, 10560) = 44.7$, $p_{GG} < 0.001$] **(Figure S1A)**. To investigate this further, a two-way ANOVA was conducted on the last 10 minutes and revealed a significant Tat effect [$F(2, 264) = 7.8$, $p < 0.001$] and a GLT effect [$F(2, 264) = 235.3$, $p < 0.001$] with GLT increasing $[Ca^{2+}]_i$ levels in a concentration dependent manner and Tat significantly enhancing $Ca^{2+}]_i$ production at the highest GLT concentration (10 µM) compared to control condition ($p = 0.002$) (**Figure S1B**). Thus, these results indicate that Tat increases glutamate-induced $[Ca^{2+}]_i$ production in frontal cortex neuron cultures, specifically at high glutamate concentrations.

Supplemental Figure Captions

Supplemental Figure 1. Primary frontal cortex neuron cultures (DIV 7-11) were untreated or pre-incubated with a subthreshold concentration of Tat 50 nM before Ca^{2+} imaging began (30 min prior). (**A**) $[Ca^{2+}]_i$ levels were plotted over a 30-minute time period with GLT (0.1 – 10 µM) being applied at the 1-minute mark (arrow). Application of 10 µM GLT onto neurons caused significant increases in $[Ca^{2+}]_i$ levels. Tat exposure significantly increased excitatory response at 10 µM GLT. (**B)** The $[Ca^{2+}]_i$ levels are summarized for the last 10 minutes of calcium assessment and indicate that only GLT 10 µM significantly upregulated $[Ca^{2+}]_i$ levels with Tat exacerbating its effect. Data are mean ± SEM. Statistical significance was determined using ANOVA and Bonferroni correction where applicable. An alpha level of $p < 0.05$ was considered significant for all statistical tests. $^*p < 0.05$ vs. GLT 10 µM (PRE: Control); $^{\#}p < 0.05$ vs. GLT 10 µM (PRE: Tat 50 nM). GLT: glutamate; PRE, pretreatment.

Supplemental Figure 2. Testing arena for the odor discrimination flexibility task. Arena constructed from lightly-textured high-density polyethylene. Holding chamber lid constructed from bulletproof glass. Arena base measured 19.75 inches across with 9-inch tall walls, holding chamber measured 3 x 3 inches with 6-inch tall walls.

Supplemental Figure 1

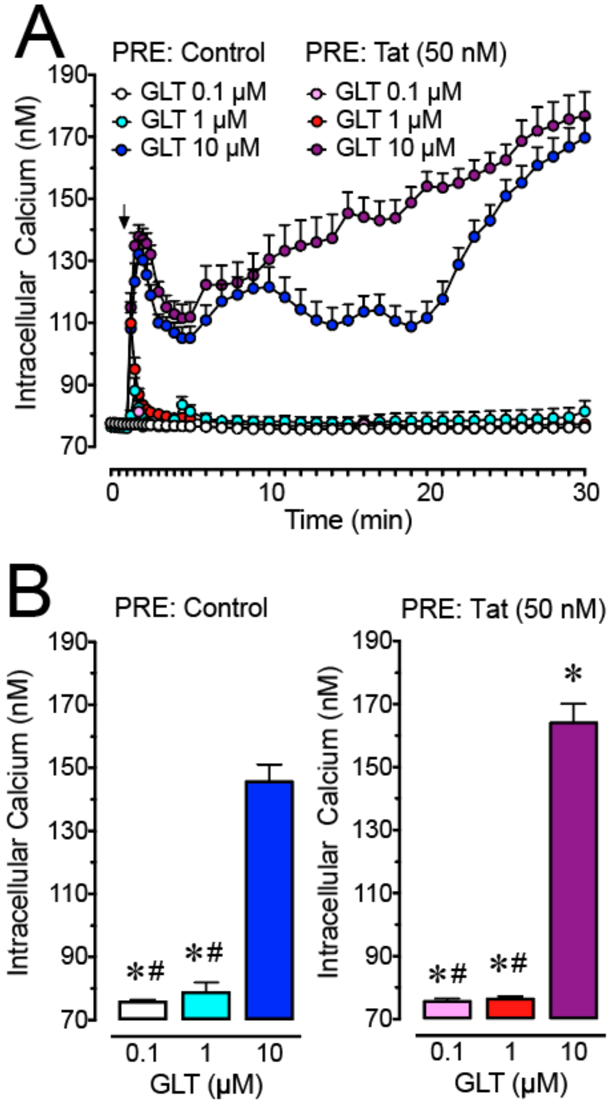

Supplemental Figure 2

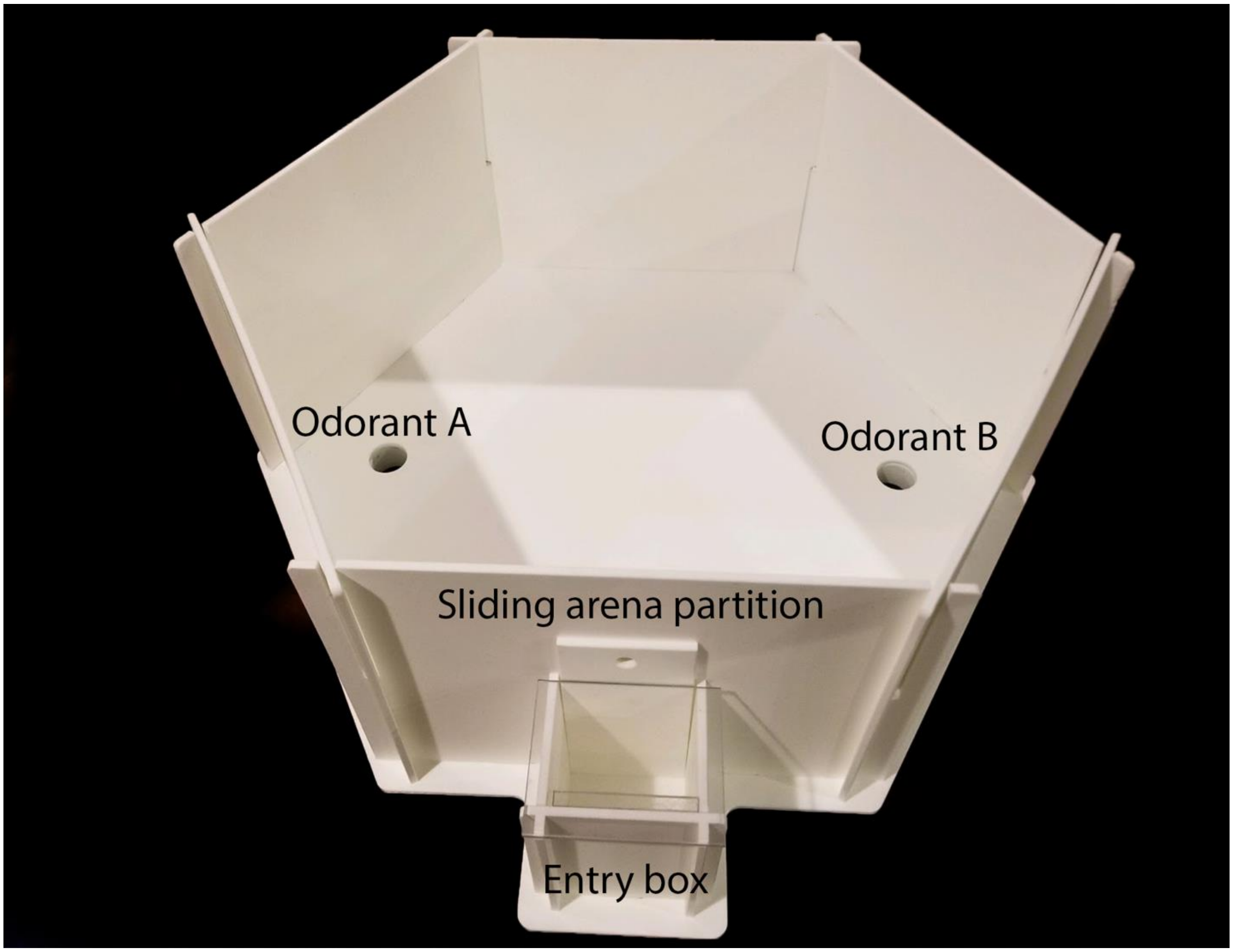

Supplement: Supplementary file 1 [file Data_Sheet_1.pdf]
